# Supplementary material for: A controlled study of emotional dysfunction in adult women with ADHD
Source: PLoS One. 2025 Dec 10;20(12):e0337454. doi: 10.1371/journal.pone.0337454 (PMC12694843; doi:10.1371/journal.pone.0337454)
Supplement: S1 Text — (DOCX) [file pone.0337454.s001.docx]

**Table A**

*Participants' Background Characteristics*

|  |  | Diagnosed ADHD * (n=70) | | Probable ADHD** (n=42) | | No ADHD***  (n=64) | | Group Differences |
| --- | --- | --- | --- | --- | --- | --- | --- | --- |
|  |  | *M* | *S.D* | *M* | *S.D* | *M* | *S.D* |  |
| Age |  | 26.29 | 2.43 | 25.69 | 3.15 | 25.53 | 2.74 | *F* (2,175) = 1.40, *p* = .25 |
| Education |  | 13.97 | 2.20 | 14.44 | 2.30 | 13.89 | 2.14 | *F* (2,151) = 0.72, *p* = .49 |
|  |  | *N* | *%* | *N* | *%* | *N* | *%* |  |
| Employment rates | *Employed* | 49 | 70 | 35 | 83.3 | 49 | 76.6 | *ꭕ2 (*2) = 0.581*, p = .*275 |
|  | *Unemployed* | 21 | 30 | 7 | 16.7 | 15 | 23.4 |  |

* Scored above the clinical cut-off point in the Adult ADHD Self-Report Scale and had a known diagnosis of ADHD.

(ASRS; Kessler et al., 2005).

** Thirty women scored above the cut-off point but were not officially diagnosed with ADHD, and 12 women scored below the cut-off point but reported a known diagnosis of ADHD.

*** Scored below the cut-off point on the ASRS and didn't have ADHD diagnosis.

**Table B**

*Descriptive Statistics*

|  | Skewness  (std. error of  skewness) | Kurtosis  (std. error  of kurtosis) | Levene's test  (based on mean)  df (1,173) |
| --- | --- | --- | --- |
| ADHD | 0.04 (0.18) | -0.72 (0.37) | 1.16, *p* = .32 |
| Working memory | -0.05 (0.18) | -1.03 (0.37) | 0.65, *p* = .53 |
| Task shifting | -0.18 (0.18) | -0.53 (0.37) | 0.63, *p* = .54 |
| ED | 0.44 (0.18) | -0.30 (0.37) | 0.91, *p* = .40 |
| Alexithymia | 0.06 (0.18) | -0.12 (0.37) | 1.45, *p* = .24 |
| Positive affect | -0.01 (0.18) | -0.24 (0.37) | 0.40, *p* =.68 |
| Negative affect | 0.27 (0.18) | -0.03 (0.37) | 1.62, *p* = .20 |

ADHD symptoms as measured by the ASRS. Working M = working memory deficits as measured by the BRIEF-A. Task Shifting, difficulty with shifting attention from one task to another as measured by the BRIEF-A; ED emotion dysregulation as measured by the DERS; Alexithymia as measured by the TAS-20; positive and negative affect as measured by the PANAS.

**Table C**

*Correlations Between Study Variables*

|  | ADHD | Working  memory | Task Shifting | ED | Alexithymia | Positive  affect | Negative  affect |
| --- | --- | --- | --- | --- | --- | --- | --- |
| ADHD | 1 | .76*** | .52*** | .57*** | .42*** | -.15 | .42*** |
| Working  memory |  | 1 | .62*** | . 55*** | .48*** | -.21** | .40*** |
| Task  shifting |  |  | 1 | . 55*** | .41*** | -.21** | .46*** |
| ED |  |  |  | 1 | .43*** | -.28*** | .56*** |
| Alexithymia |  |  |  |  | 1 | -.17* | .44*** |
| Positive  affect |  |  |  |  |  | 1 | -0.21** |
| Negative  affect |  |  |  |  |  |  | 1 |

Notes: **p* < .05, ***p* < .01, *** *p* < .001. ADHD symptoms as measured by the ASRS. Working M = working memory deficits as measured by the BRIEF-A. Task Shifting, difficulty with shifting attention from one task to another as measured by the BRIEF-A; ED emotion dysregulation as measured by the DERS; Alexithymia as measured by the TAS-20; positive and negative affect as measured by the PANAS.

**Table D**

*Linear Regression Predicting Emotional Dysregulation*

|  |  | *Beta* | *t* | *p* | *R^2^* |
| --- | --- | --- | --- | --- | --- |
| Step 1 | *F*_(2,151)_ = 0.91, *p* = 0.406, *R^2^* = 0.01, *p* < .001 | | | | |
|  | Age | -.01 | -.14 | .885 | -.001 |
|  | Education | -.10 | -.98 | .328 |  |
| *Step 2* | *F*_(3,151)_ = 23.40, *p* < .001, *R^2^* = 0.32, *R^2^* change = 0.31 | | | | |
|  | Age | -.12 | -1.41 | .162 | 0.31 |
|  | Education | -.01 | -.06 | .949 |  |
|  | ADHD | .56 | 8.22 | <.001 |  |
| *Step 3* | *F*_(4,151)_ = 21.63, *p* < .001, *R^2^* = 0.37, *R^2^* change = .05 | | | | |
|  | Age | -.11 | -1.37 | .172 | 0.37 |
|  | Education | -.02 | -.24 | .811 |  |
|  | ADHD | .30 | 2.90 | .004 |  |
|  | Working  Memory  deficits | .34 | 3.37 | <.001 |  |
|  |  |  |  |  |  |
| Step 1 | *F*_(2,151)_ = 0.91, *p* = 0.406, *R^2^* = 0.01 | | | | |
|  | Age | -.01 | -.14 | .885 | -.001 |
|  | Education | -.10 | -.98 | .328 |  |
| *Step 2* | *F*_(3,151)_ = 23.40 , *p* < .001, *R^2^* = 0.32, *R^2^* change = 0.31 | | | | |
|  | Age | -.12 | -1.41 | .162 | 0.31 |
|  | Education | -.01 | -.06 | .949 |  |
|  | ADHD | .56 | 8.22 | <.001 |  |
| *Step 3* | *F*_(4,151)_ = 27.00, *p* < .001, *R^2^* = 0.42, *R^2^* change = .10 | | | | |
|  | Age | -.08 | -1.03 | .306 | 0.41 |
|  | Education | -.01 | -.14 | .889 |  |
|  | ADHD | .35 | 4.71 | <.001 |  |
|  | Task shifting  deficits | .38 | 5.09 | <.001 |  |

**Figure A**.

*Difficulties in emotion regulation and Executive Functions*


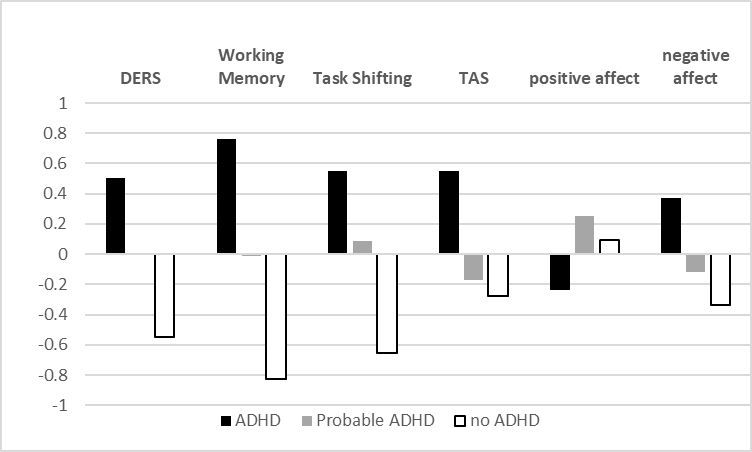


DERS=Difficulties in Emotion Regulation (Gratz & Roemer, 2004); (Difficulties in) Working Memory and Task Shifting as measured by the BRIEF-A (Roth et al., 2005); TAS= Alexithymia as measured by the TAS-20 (Bagby et al., 1994); Positive and Negative Affect as measured by the PANAS (Watson et al., 1988).

**Figure B**

*Executive Function Deficits Mediate the Relationship between ADHD Symptoms and Emotional Dysregulation.*

Task Shifting

b1 = .38***

a1 = .55***

c1 = .56***; c1’ = .35***

Emotional Dysregulation

ADHD

c2 = .56***; c2’ = .30**

a2 = .77***

b2 = .34***

Working Memory

Notes: **p* < .05, ***p* < .01, *** *p* < .001. Numbers represent β values. ADHD symptoms as measured by the ASRS. Working Memory = working memory deficits as measured by the BRIEF-A. Task Shifting, difficulty with shifting attention from one task to another as measured by the BRIEF-A; Emotion Dysregulation as measured by the DERS.
