# Supplementary material for: A controlled study of emotional dysfunction in adult women with ADHD
Source: PLoS One. 2025 Dec 10;20(12):e0337454. doi: 10.1371/journal.pone.0337454 (PMC12694843; doi:10.1371/journal.pone.0337454)
Supplement: Attachment — Submitted filename: Review A Controlled Study of Emotional Dysfunction....docx [file pone.0337454.s003.docx]

Review A Controlled Study of Emotional Dysfunction in Adult Women with ADHD

I think the paper is relevant, about a relevant topic and still not sufficiently researched. It is well written and organized. However, the following features should be improved.

**Introduction**:

P.5, line 115: “The second model perceives ADHD with emotional dysregulation as qualitatively different from pure ADHD, which has a distinct etiological entity and ADHD course”.- There is no reference for this model.

**Methods:**

P.9, line 205: “All participants signed a written informed consent”. – By the position this statement is in the text, it seems only the first group (women with ADHD) signed this consent.

P.10, line 225: “The three groups were found to have very similar characteristics, and were not significantly different in age, years of education and employment”.- The variable “employment” is not present in the preious sample characterization, the mean age and SD are also absent…a table with all the information about the sample should be presented.

P.12, line 285: “In the current study, the reliability of the subscales was .88, .80, and .59…”.- this last value is not acceptable, and the authors have already removed a subscale of the Emotion Regulation scale due to a value of .57. I think this should be removed too.

P.13, line 296: Data Analysis: The analysis on the normality of distribution and homogeneity of variance should be presented.

P.13, line 298: “To examine the second hypothesis, we conducted a Univariate Analysis of Variance with post-hoc comparisons using the Bonferroni range correction”. – why didn´t the authors use MANOVA? That’s the adequate analysis for multiple dependent variables and protects against Type I errors; ANOVA has Bonferroni correction for post-hoc tests only, but not for the initial composite.

**Discussion**:

The authors do not discuss results that were not expected. Namely, the fact that positive affect does not correlate with ADHD symptoms severity, and that there are no significant differences in positive affect between Groups 1 and 3.

P.16, line 370: “emotional **liability**” – typo.

P.17, limitations: there is, in my view, a major limitation that the authors did not state: the executive functions should not be accessed by self-report, but by performance-based tests.

P.17, line e 410: “Third, reliance on self-report measures may involve problems of shared method variance”.- The authors can assess this with statistical methods, I recommend this.

P.18, line 427: Shifting clinical attention to emotional dysregulation symptoms, rather than core ADHD symptoms of hyperactivity and impulsivity which are usually less dominant in women, may improve identification and intervention”.- I agree with the fact that it may improve intervention. However, how can it improve identification of ADHD? By itself, it can´t, because emotional dysregulation is present in several disorders…; the authors should reflect on this and reframe.
